# Modeling and validation of serum miR-18a and miR-122 levels as predictors of recurrence after laparoscopic radical cystectomy procedure for bladder cancer based on nomogram model

**DOI:** 10.3389/fonc.2025.1579873

**Published:** 2026-01-09

**Authors:** Peng Wang, Jun Deng, Shuai Wu

**Affiliations:** Department of Urology Surgery, Qingdao Hospital, University of Health and Rehabilitation Sciences (Qingdao Municipal Hospital), Qingdao, China

**Keywords:** bladder cancer, laparoscopic radical cystectomy, nomogram prediction model, serum miR-122, serum mir-18a

## Abstract

**Objective:**

This study aimed to identify factors influencing recurrence after laparoscopic radical cystectomy for bladder cancer (BC) based on serum levels of miR-18a and miR-122, and to develop and validate a nomogram prediction model.

**Methods:**

The relevant information of BC patients who received laparoscopic radical cystectomy procedure in our hospital from January 2021 to October 2022 was collected retrospectively. The patients were divided into a training set and a validation set at a ratio of 7:3 by the complete randomization method. Independent predictive variables included in the Nomogram model were determined and modeled through univariate analysis and multivariate Logistic regression analysis. The receiver operating characteristic curve (ROC) and calibration curves were used to evaluate the predictive efficacy of the model, and decision curve analysis (DCA) was used to evaluate its clinical application value.

**Results:**

A total of 280 research subjects were included. Recurrence occurred in 46 (23.47%) of the 196 patients in the training set and 21 (25.00%) of the 84 patients in the validation set. The results of the multivariate logistic regression analysis showed that preoperative serum miR-18a levels, preoperative serum miR-122 levels, postoperative serum carcinoembryonic antigen levels, postoperative serum carbohydrate antigen 19–9 levels, and postoperative antibiotic use duration were significantly associated with recurrence after laparoscopic radical cystectomy for BC. The model was well calibrated and fitted in the training and validation sets. The ROC curve showed that the AUC of the nomogram model to predict postoperative recurrence were 0.796(95% CI: 0.688-0.904) and 0.762(95% CI: 0. 578-0.946), respectively. DCA indicated that the model had clinical application value.

**Conclusion:**

The Nomogram model for recurrence after laparoscopic radical cystectomy procedure for BC has good prediction ability.

## 1 Introduction

Bladder cancer (BC) is a common and extremely dangerous malignant tumor in the urinary system, which originates from the malignant transformation and abnormal proliferation of bladder mucosal epithelial cells to form a lesion (1, 2). Clinically, there are various treatments for BC, and laparoscopic radical cystectomy procedure is one of the important treatments, which has the advantages of relatively small trauma and rapid recovery after surgery (3). However, even after surgical resection, BC still has a certain risk of recurrence. Accurate prediction of BC postoperative recurrence is particularly crucial for optimizing the treatment plan and improving the prognosis of patients.

As cancer research advances, biomarkers have gained significant interest. Serum microRNA (miRNA), in particular, has attracted researchers’ attention due to its stability and ease of detection (4). Among them, serum miR-18a is found to be widely involved in the process of cell proliferation, differentiation, apoptosis, and others. Studies have revealed that it plays an important role in the occurrence and development of a variety of tumors, which is closely related to the invasion and metastasis potential of tumor cells and is expected to become a key indicator for early diagnosis and prognosis judgment of tumors (5–7). However, miR-122 has been studied for a long time in the field of liver-related diseases. Recent evidence indicates that miR-122 also has a unique regulatory function in urinary system tumors, especially in BC, that it can affect the biological behavior of BC, or serve as a potential marker reflecting the progression of BC’s disease (8–10).

In view of this, the purpose of this study was to comprehensively consider the serum levels of miR-18a and miR-122, explore the influencing factors of recurrence after laparoscopic radical cystectomy procedure for BC, and construct and verify Nomogram prediction model based on these factors, in the hope of providing a powerful reference for clinical decision-making and assisting the accurate diagnosis and treatment of BC patients.

## 2 Data and methods

### 2.1 Study objects

**Figure 1:**
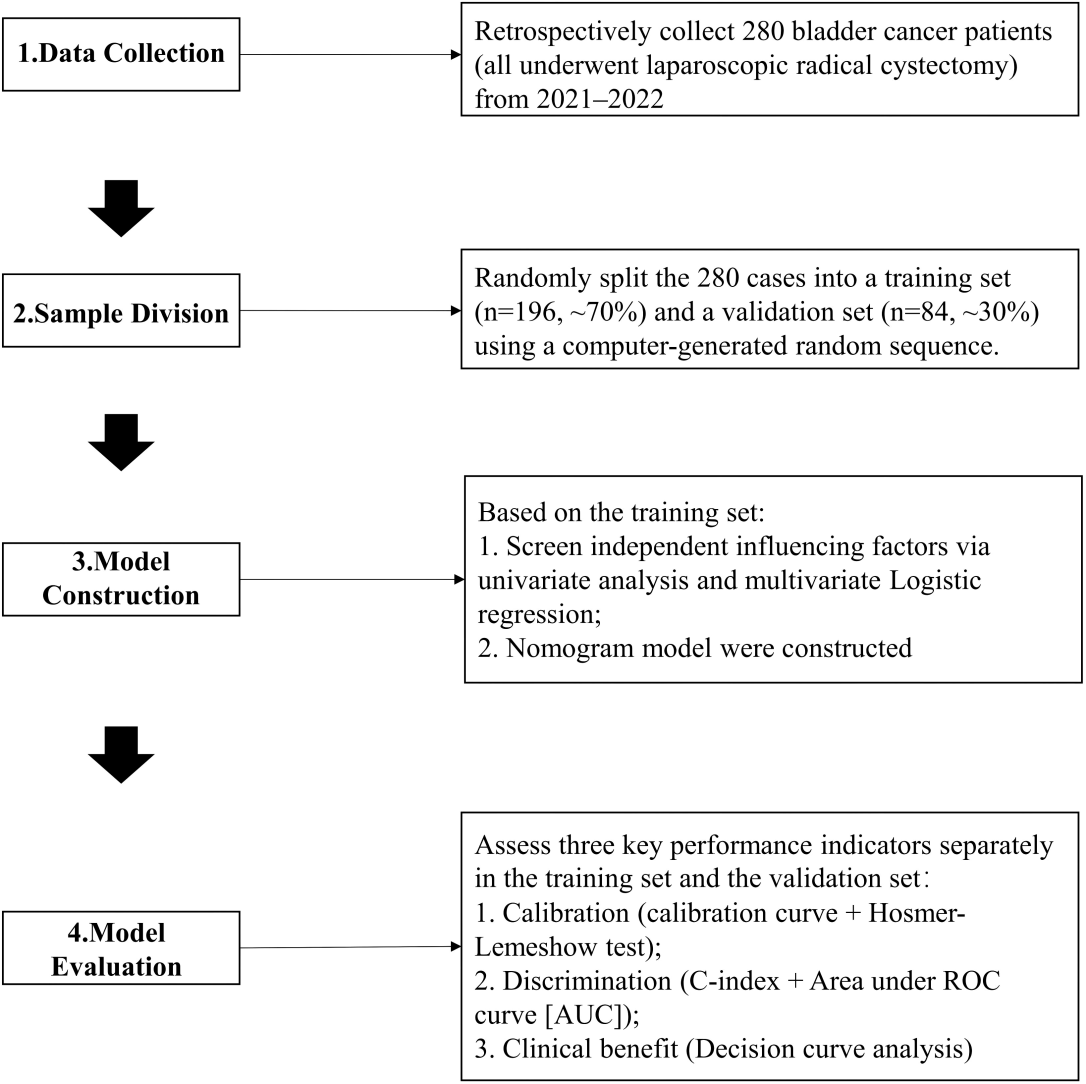
Flow chart for construction of a nomogram model for predicting recurrence after laparoscopic radical cystectomy in bladder cancer.

Following approval from our hospital’s Ethics Committee, we retrospectively enrolled 280 BC patients who underwent laparoscopic radical cystectomy between January 2021 and October 2022. Patients were randomly assigned to a training set and a validation set in a 7:3 ratio. This ratio was chosen to balance the need for sufficient data for model training against the requirement for an adequately sized independent set for reliable performance validation, a common practice in machine learning-based clinical research (11, 12). The final group sizes (196 patients in the training set and 84 patients in the validation set) exactly match the intended 7:3 distribution. The training set was used to identify risk factors and construct the nomogram prediction model. The validation set was used to provide an initial, independent assessment of the model’s discrimination, calibration, and clinical usefulness. All participants were informed and agree. The specific research methods and steps were shown in **Figure 1**.

### 2.2 Inclusion exclusion criteria

#### 2.2.1 Inclusion criteria

(1) BC is confirmed by histopathological examination, covering urothelial carcinoma, squamous cell carcinoma, adenocarcinoma and other pathological types, and it is determined based on standard pathological report. (2) Adult patients at the age of 18 years or above are ensured to cooperate with the follow-up examination, treatment and follow-up. (3) Laparoscopic radical cystectomy procedure is adopted. (4) Patients with 0–2 scores were selected according to the Eastern Cooperative Oncology Group (ECOG) physical status score. They had basic self-care ability, and tolerated surgery and regular follow-up.

#### 2.2.2 Exclusion criteria

(1) Patients with a history of other serious malignant tumors that are not effectively controlled, such as advanced lung cancer and the stage of extensive metastasis of liver cancer. (2) Complicated with severe cardiac, hepatic and renal insufficiency, such as chronic renal failure requiring long-term dialysis, severe heart failure NYHA grade III-IV, decompensated liver cirrhosis, etc. (3) Mental disease or cognitive disorder cannot cooperate with the questionnaire, regular blood collection and test, and timely follow-up. (4) Patients who have received other anti-cancer treatments for BC, such as chemoradiotherapy before surgery.

### 2.3 Laparoscopic radical cystectomy procedure treatment

The surgical procedure involves multiple steps. Before the operation, the patient was under general anesthesia, put in the supine position, and disinfected the drape. After an incision was made at the navel, a pneumoperitoneum needle was inserted, and carbon dioxide gas was injected to prop up the abdominal cavity. Then the laparoscope was inserted. In addition, two to three auxiliary operation channels were established at appropriate sites in the abdomen. During the operation, ultrasonic scalpel was used to separate the ligaments and tissues around the bladder. For men, the prostate needed to be treated, and for women, operations related to the anterior vaginal wall were involved. Meanwhile, the ureter was freed and cut for marking. A standard pelvic lymph node dissection (pLND) was routinely performed for all patients, which included the removal of nodal tissue from the obturator, internal iliac, and external iliac regions. Subsequently, the bladder was completely removed. The specimens were packaged and taken out through the enlarged incision. After complete removal of the bladder, intraoperative frozen section analysis was routinely performed on the distal ureteral margins to ensure negative surgical margins. In cases where an orthotopic neobladder was planned, frozen section analysis of the proximal urethral margin was also carried out. If a positive margin was identified, additional tissue was resected until a negative margin was confirmed. Final pathological examination of all margins was performed postoperatively on the permanent sections. Finally, according to the patient’s situation, either an ileal conduit (Bricker procedure) or an orthotopic neobladder procedure was selected for urinary diversion. Postoperative recovery was managed according to our institutional protocol. Based on anesthesiologist’s recommendation and patient’s comorbidity status, selective patients, particularly those with significant comorbidities or intraoperative instability, were admitted to the Intensive Care Unit for close monitoring immediately after surgery and transferred to the general ward once stabilized. Other patients with stable conditions were directly transferred to the specialized urology ward with continuous monitoring capabilities. Adequate analgesia and wound care were provided throughout. Fasting and intravenous nutrition were provided before the recovery of intestinal function, and diet was gradually restored after the restoration of exhaust and defecation. In terms of urinary tract, patients undergoing ileal conduit need to learn ostomy nursing, while patients undergoing orthotopic neobladder need to train bladder function and pay attention to the presence of abnormal urination, in order to ensure a good prognosis for patients.

### 2.4 Preoperative investigation

#### 2.4.1 Questionnaire

Patients’ basic information (age, gender, body mass index (BMI), smoking history, drinking history, occupational exposure history, BC family history) and comorbidities (hypertension and diabetes) were investigated by questionnaire before surgery.

#### 2.4.2 Cystoscopy

The diagnosis and initial pathological type of bladder cancer were confirmed via cystoscopic biopsy prior to surgery. This preoperative pathological confirmation was the basis for patient inclusion and surgical decision-making. The definitive pathological staging was obtained from the analysis of the radical cystectomy specimens postoperatively.

#### 2.4.3 CT examination

The TNM staging system (union for international cancer control, UICC) (13) was adopted to assist in the determination of T, N and M stages by examining the tumor size and location, whether it invades the surrounding tissues, and whether there is lymph node metastasis and distant metastasis through CT examination.

The World Health Organization (WHO) grading system (14) was adopted to observe the differentiation degree of tumor cells, nuclear atypia and mitotic figures. The morphology of well-differentiated tumor cells is close to that of normal cells, while that of poorly differentiated tumor cells are characterized by high pleomorphism and abundant mitotic figures, thus judging the grade.

#### 2.4.4 Serum examination

Peripheral venous blood was collected from the patient. A conventional venipuncture was performed and an appropriate amount of blood was drawn from the arm vein into a blood collection tube containing an anticoagulant in the fasted state of the patient. The blood collection volume was about 3–5 mL.

MiR-18a and miR-122 were extracted using the miRNeasy Serum/Plasma Kit (Qiagen, Hilden, Germany) according to the manufacturer’s operating procedures. U6 small nuclear RNA (U6 snRNA) was used as the internal reference gene. Reverse transcription was performed using the TaqMan MicroRNA Reverse Transcription Kit (Applied Biosystems, Foster City, USA) under the following conditions: 16 °C for 30 min, 42 °C for 30 min, and 85 °C for 5 min. For qPCR amplification, specific TaqMan probes and primers (Applied Biosystems, Cat. No.: hsa-miR-18a-5p: 4427975; hsa-miR-122-5p: 4427975; U6 snRNA: 4427975) were used, with the reaction system (20 μL) containing 10 μL of TaqMan Universal PCR Master Mix, 1 μL of cDNA template, 1 μL of primer-probe mix, and 8 μL of nuclease-free water. The qPCR conditions were: 95 °C for 10 min, followed by 40 cycles of 95 °C for 15 s and 60 °C for 1 min. Negative controls (nuclease-free water instead of cDNA template) and positive controls (commercially available miR-18a/miR-122 standard solutions) were included in each run to ensure assay specificity and reliability. Raw qPCR data (Ct values) have been provided in the supplementary materials. Absolute quantification of serum miR-18a and miR-122 levels (expressed as pg/mL) was achieved by constructing standard curves using serial dilutions of synthetic miR-18a and miR-122 standard substances (Thermo Fisher Scientific, Waltham, USA). The standard curves were established by plotting the logarithm of the standard concentration against the corresponding Ct values, and the concentrations of target miRs in samples were calculated based on the linear regression equation derived from the standard curves.

### 2.5 Postoperative investigation

#### 2.5.1 Serum test

Peripheral venous blood was collected from the patient within 1–3 days after surgery (early postoperative period, prior to the formal follow-up) in an amount of 3 to 5 mL. The blood samples were placed in a centrifuge and centrifuged at 1000-3000 (rpm) for 10–15 minutes to separate the blood layers. The serum is then transferred by pipette to a clean centrifuge tube. Reaction tubes containing serum samples were placed in a Cobas e601 automatic chemiluminescent immunoassay analyzer (Roche Diagnostics, Basel, Switzerland). Specific carcinoembryonic antigen (CEA) and carbohydrate antigen 19-9 (CA19-9) antibodies were provided in the Elecsys CEA Kit (Cat. No.: 05938332190) and Elecsys CA19–9 Kit (Cat. No.: 05938364190, both from Roche Diagnostics, Basel, Switzerland), respectively, and added according to the kit instructions. Then after a washing step, unbound substances are removed, and a luminescent substrate is added to initiate a chemiluminescent reaction. The instrument detects the intensity of the luminescent signal and calculates the levels of CEA and CA19–9 in serum based on a pre-established standard curve.

#### 2.5.2 Postoperative observation

Postoperative medical staff should closely observe and record the specific hours of first urination after operation. For indwelling catheter, to pay attention to the insertion and extraction time, in order to accurately grasp the indwelling days; At the same time, strictly follow the doctor’s advice medication process, record the date of antibiotics began to use and stop using, calculate the duration of use. During the observation, if abnormal data or unclear records are found, the medical staff shall timely communicate and check the data, and further understand the details from the patients and their families when necessary to ensure the accuracy and reliability of the data.

### 2.6 Follow-up survey

#### 2.6.2 Follow-up time and content

The follow-up period was 28 months. Patients were regularly followed up through clinical visits and telephone interviews. Recurrence was monitored primarily using imaging examinations, such as computed tomography (CT) or magnetic resonance imaging (MRI) of the abdomen and pelvis, which were performed at scheduled intervals. Information on adjuvant therapies (chemotherapy or radiotherapy) was collected from hospital medical records. The adjuvant chemotherapy regimens mainly included gemcitabine plus cisplatin (GC regimen) and methotrexate + vinblastine + doxorubicin + cisplatin (MVAC regimen).

#### 2.6.2 Recurrence determination method

During the follow-up period, regular imaging examinations such as CT or MRI of the pelvis and abdomen were performed. The occurrence of local recurrence or distant metastasis was assessed based on imaging findings. If suspicious lesions were identified, a biopsy was performed. Pathological results confirming the presence of malignant tumor tissue consistent with bladder cancer were judged as relapse.

### 2.7 Statistical analysis

Statistical analysis was performed using SPSS 26.0 and R 4.2.3. The enumeration data were described by frequency and percentage, and *χ*^2^ test was used. Measurement data of normal distribution were expressed with mean standard deviation, and independent sample t test was used. In the training set, univariate analysis was conducted for all variables to initially screen out variables that might be associated with the outcome. The variables selected through univariate analysis were then included in multivariate Logistic regression analysis to further identify the independent risk factors (*P*<0.05), and their odds ratios (OR) and 95% confidence intervals (CI) were calculated. The receiver operating characteristic (ROC) curve of the model was drawn to determine the sensitivity, specificity, Youden’s index and optimal cutoff value of the model. The model discrimination was assessed by area under the curve (AUC). The larger the value was, the better the discrimination was. A calibration curve was plotted and evaluated using the Hosmer-Lemeshow test. The Decision curve analysis (DCA) was drawn to test the actual application effectiveness of the model. A *P* value < 0.05 was considered statistically significant.

## 3 Results

### 3.1 Comparison of baseline characteristics between the training set and the validation set

A total of 280 BC patients who received laparoscopic radical cystectomy procedure were included. Recurrence occurred in 46 (23.47%) of the 196 patients in the training set and 21 (25.00%) of the 84 patients in the validation set. There was no significant difference in the recurrence rate, serum miR-18a, miR-122 levels and other related parameters between the training set and the validation set (*P* > 0.05) (**Table 1**).

**Table 1:** Comparison of baseline characteristics between the training set and the validation set.

Indicators			Training set (n=196)	Validation set (n=84)	Statistical values	*P*
Age (years)			59.21 ± 8.18	61.25 ± 8.44	1.890	0.060
Gender	Male		107(54.59)	52(61.90)	1.281	0.258
	Female		89(45.41)	32(38.10)		
BMI (kg/m^2^)			23.93 ± 2.61	24.21 ± 2.42	0.838	0.403
Hypertension	Yes		61(31.12)	35(41.67)	2.902	0.088
	No		135(68.88)	49(58.33)
Diabetes	Yes		65(33.16)	31(36.90)	0.365	0.546
	No		131(66.84)	53(63.10)
Smoking history	Yes		95(48.47)	50(59.52)	2.878	0.090
	No		101(51.53)	34(40.48)
Drinking history	Yes		105(53.57)	41(48.81)	0.534	0.465
	No		91(46.43)	43(51.19)
Occupational exposure history	Yes		60(30.61)	29(34.52)	0.415	0.519
	No		136(69.36)	55(65.48)
BC family history	Yes		22(11.22)	15(17.86)	2.256	0.133
	No		174(88.78)	59(70.24)
Pathological type of tumor	Urothelial carcinoma		163(83.16)	62(73.81)	3.536	0.316
	Squamous cell carcinoma		22(11.22)	16(19.05)
	Glandular cancer		7(3.57)	4(4.76)
	Other		4(2.04)	2(2.38)
Tumor staging	Tis-T1		76(38.78)	36(42.86)	1.066	0.785
	T2		65(33.16)	29(34.52)
	T3		38(19.39)	14(16.67)
	T4		17(8.67)	5(5.95)
Tumor grading	Low-grade		40(20.41)	21(25.00)	0.728	0.394
	High-grade		156(79.59)	63(75.00)
Preoperative Serum miR-18a Levels (pg/mL)			8.75 ± 3.02	8.21 ± 3.11	1.376	0.170
Preoperative Serum miR-122 Levels (pg/mL)			7.00 ± 2.25	7.35 ± 2.46	1.169	0.243
Postoperative serum CEA level (ng/mL)			3.61 ± 1.26	3.32 ± 1.31	1.769	0.078
Postoperative serum CA19–9 levels (U/mL)			23.87 ± 7.23	24.81 ± 8.54	0.884	0.378
Postoperative first urination time (h)			7.90 ± 2.43	8.21 ± 2.75	0.943	0.347
Postoperative indwelling catheter time (d)			3.91 ± 1.38	3.74 ± 1.52	0.915	0.361
Postoperative antibiotic use time (d)			5.47 ± 1.76	5.21 ± 1.84	1.094	0.275
Are you receiving adjuvant chemotherapy after surgery		Yes	75(38.27)	23(27.38)	3.062	0.080
		No	121(61.73)	61(72.62)
Do you receive adjuvant radiotherapy after surgery		Yes	52(26.53)	20(23.81)	0.228	0.633
		No	144(73.47)	64(76.19)

To evaluate the potential impact of adjuvant chemotherapy on postoperative biomarker levels, we compared serum CEA and CA19–9 levels between patients who received adjuvant chemotherapy and those who did not. The results showed that the postoperative serum CEA level was 3.52 ± 1.31 ng/mL in the adjuvant chemotherapy group and 3.47 ± 1.28 ng/mL in the non-adjuvant chemotherapy group (*P* = 0.321). The postoperative serum CA19–9 level was 24.12 ± 7.85 U/mL in the adjuvant chemotherapy group and 23.76 ± 7.43 U/mL in the non-adjuvant chemotherapy group (*P* = 0.285). There were no significant differences in postoperative CEA and CA19–9 levels between the two groups, suggesting that adjuvant chemotherapy had limited confounding effects on these biomarkers in the current cohort.

### 3.2 Univariate analysis between the relapsed group and the non-relapsed group in the training set

In the training set, univariate analysis showed that there were significant differences in preoperative serum miR-18a level, preoperative serum miR-122 level, postoperative serum CEA level, postoperative serum CA19–9 level, and postoperative antibiotic use time between the relapsed group and the non-relapsed group (all *P* < 0.05) (**Table 2**).

**Table 2:** Comparison of serum miR-18a and miR-122 levels and other related parameters between the relapsed group and the non-relapsed group in the training set.

Indicators		Recurrence group (n=46)	Non-recurrence group (n=150)	Statistical values	*P*	OR	95%
Age (years)		60.52 ± 8.25	58.20 ± 7.91	1.723	0.087	1.038	0.995-1.083
Gender	Male	29(63.04)	78(52.00)	1.732	0.188	1.575	0.799-3.105
	Female	17(36.96)	72(48.00)				
BMI (kg/m^2^)		24.12 ± 2.25	23.87 ± 2.71	0.590	0.556	1.040	0.914-1.182
Hypertension	Yes	19(41.30)	42(28.00)	2.907	0.088	1.810	0.911-3.596
	No	27(58.70)	108(72.00)				
Diabetes	Yes	11(23.91)	54(36.00)	2.320	0.128	0.559	0.263-1.189
	No	35(76.90)	96(64.00)				
Smoking history	Yes	27(58.70)	68(45.33)	1.589	0.114	0.714	0.878-3.346
	No	19(41.30)	82(54.67)				
Drinking history	Yes	24(52.17)	81(54.00)	0.047	0.828	0.929	0.480-1.801
	No	22(47.83)	69(46.00)				
Occupational exposure history	Yes	17(36.96)	43(28.67)	1.139	0.286	1.459	0.728-2.924
	No	29(63.04)	107(71.33)				
BC family history	Yes	6(13.04)	16(10.67)	0.200	0.655	1.256	0.461-3.423
	No	40(86.96)	134(89.33)				
Pathological type of tumor	Urothelial carcinoma	36(78.26)	127(84.67)	1.134	0.769	1.209	0.734-1.991
	Squamous cell carcinoma	7(15.22)	15(10.00)				
	glandular cancer	2(4.35)	5(3.33)				
	other	1(2.17)	3(2.00)				
Tumor staging	Tis-T1	13(28.26)	63(42.00)	2.944	0.400	1.304	0.933-1.823
	T2	17(36.96)	48(32.00)				
	T3	11(23.91)	27(18.00)				
	T4	5(10.87)	12(8.00)				
Tumor grading	Low-grade	7(15.22)	33(22.00)	0.997	0.318	1.373	0.853-2.202
	High-grade	39(84.78)	117(78.00)				
Preoperative Serum miR-18a Levels (pg/mL)		9.51 ± 3.84	8.21 ± 2.51	2.684	0.008	1.168	1.038-1.315
Preoperative Serum miR-122 Levels (pg/mL)		7.65 ± 2.21	6.50 ± 2.01	3.315	0.001	1.137	1.110-1.562
Postoperative serum CEA level (ng/mL)		4.23 ± 1.62	3.24 ± 0.82	5.538	0.001	2.321	1.618-3.331
Postoperative serum CA19–9 levels (U/mL)		25.21 ± 7.65	22.54 ± 6.57	2.318	0.022	1.060	1.008-1.115
Postoperative first urination time (h)		8.10 ± 2.21	7.85 ± 2.51	0.608	0.544	1.043	0.910-1.196
Postoperative indwelling catheter time (d)		4.20 ± 1.80	3.82 ± 1.22	1.327	0.190	1.219	0.957-1.552
Postoperative antibiotic use time (d)		6.01 ± 1.59	5.23 ± 1.54	2.982	0.003	1.365	1.099-1.696
Are you receiving adjuvant chemotherapy after surgery	Yes	12(26.09)	63(42.00)	3.774	0.052	0.487	0.234-1.015
	No	34(73.91)	87(58.00)				
Do you receive adjuvant radiotherapy after surgery	Yes	10(21.74)	42(28.00)	0.708	0.400	0.714	0.325-1.568
	No	36(78.26)	108(72.00)				

### 3.3 Multivariate logistic regression of influencing factors for recurrence after laparoscopic radical cystectomy for treatment of BC

Recurrence or not was considered as the dependent variable (0= no recurrence, 1= recurrence), and the factor with *P* < 0.05 in Univariate analysis was considered as the covariate. Further multivariate Logistic regression analysis showed that preoperative serum miR-18a level, preoperative serum miR-122 level, postoperative serum CEA level, postoperative serum CA19–9 level, and postoperative antibiotic use time were significantly associated with recurrence after laparoscopic radical cystectomy for BC (all *P* < 0.05) (**Table 3**).

**Table 3:** Multivariate logistic regression analysis of risk factors for postoperative recurrence of BC.

Factors	B	S.E.	Wald	*P*	OR	95%CI
Preoperative serum miR-18a level	0.189	0.068	7.658	0.006	1.208	1.057-1.382
Preoperative serum miR-122 level	0.263	0.100	6.858	0.009	1.301	1.068-1.584
Postoperative serum CEA levels	0.823	0.197	17.577	0.001	2.283	1.552-3.357
Postoperative serum CA19–9 level	0.062	0.030	4.233	0.040	1.064	1.003-1.130
Postoperative antibiotic use time	0.321	0.129	6.206	0.013	1.379	1.071-1.775
Constant	-10.994	1.784	37.985	0.001		

### 3.4 Development of nomogram prediction model for postoperative recurrence of BC

**Figure 2:**
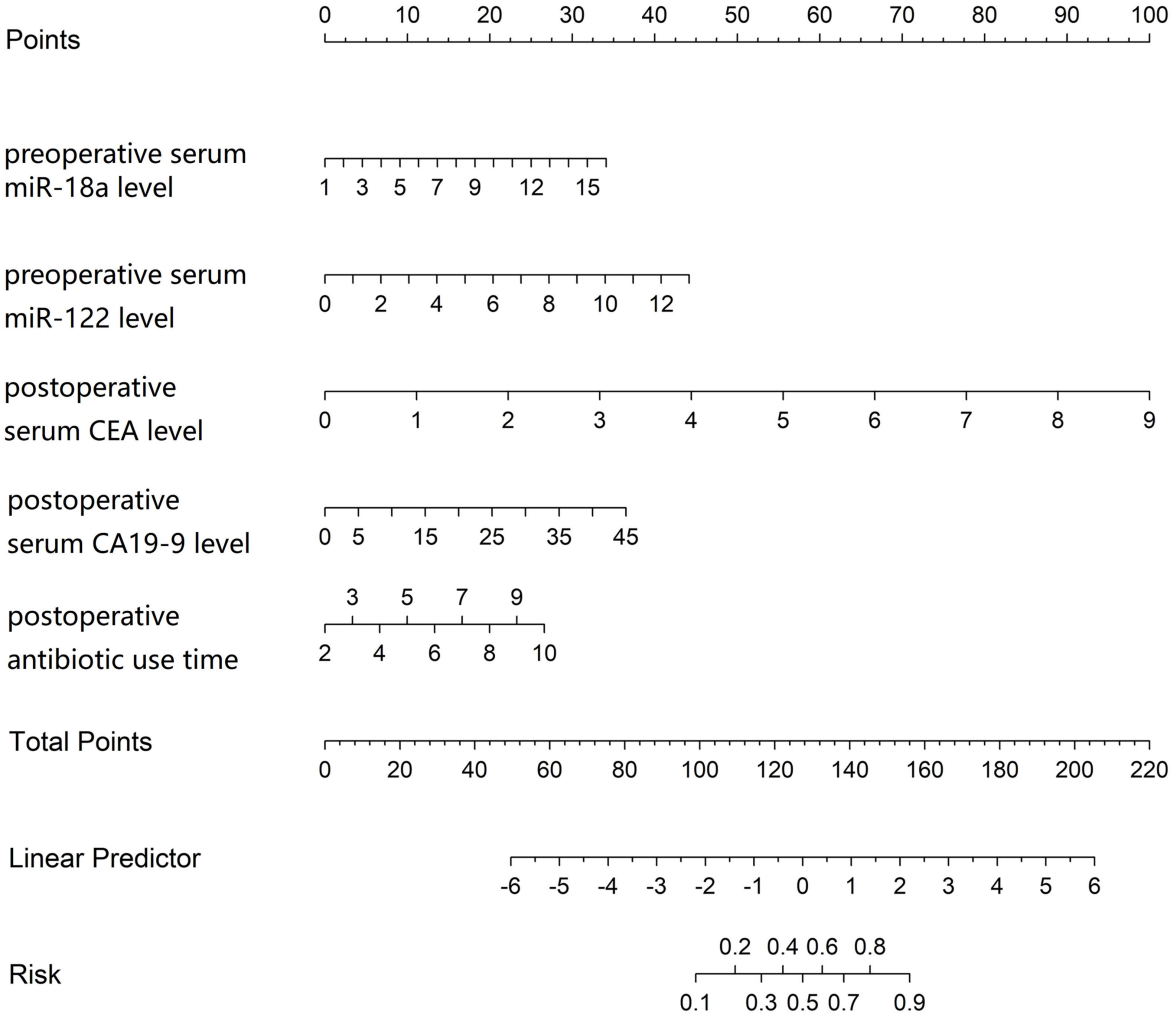
Nomogram of bladder cancer postoperative recurrence prediction model.

Based on the independent risk factors identified by multivariate Logistic regression analysis, a nomogram prediction model for BC postoperative recurrence was constructed. Each independent risk factor in the model was scored, and the total score for predicting BC postoperative recurrence was calculated, which was reflected in the prediction of BC postoperative recurrence rate. The higher the total score was, the higher the risk for BC postoperative recurrence was (**Figure 2**).

### 3.5 Evaluation and validation of nomogram prediction model for postoperative recurrence of BC

**Figure 3:**
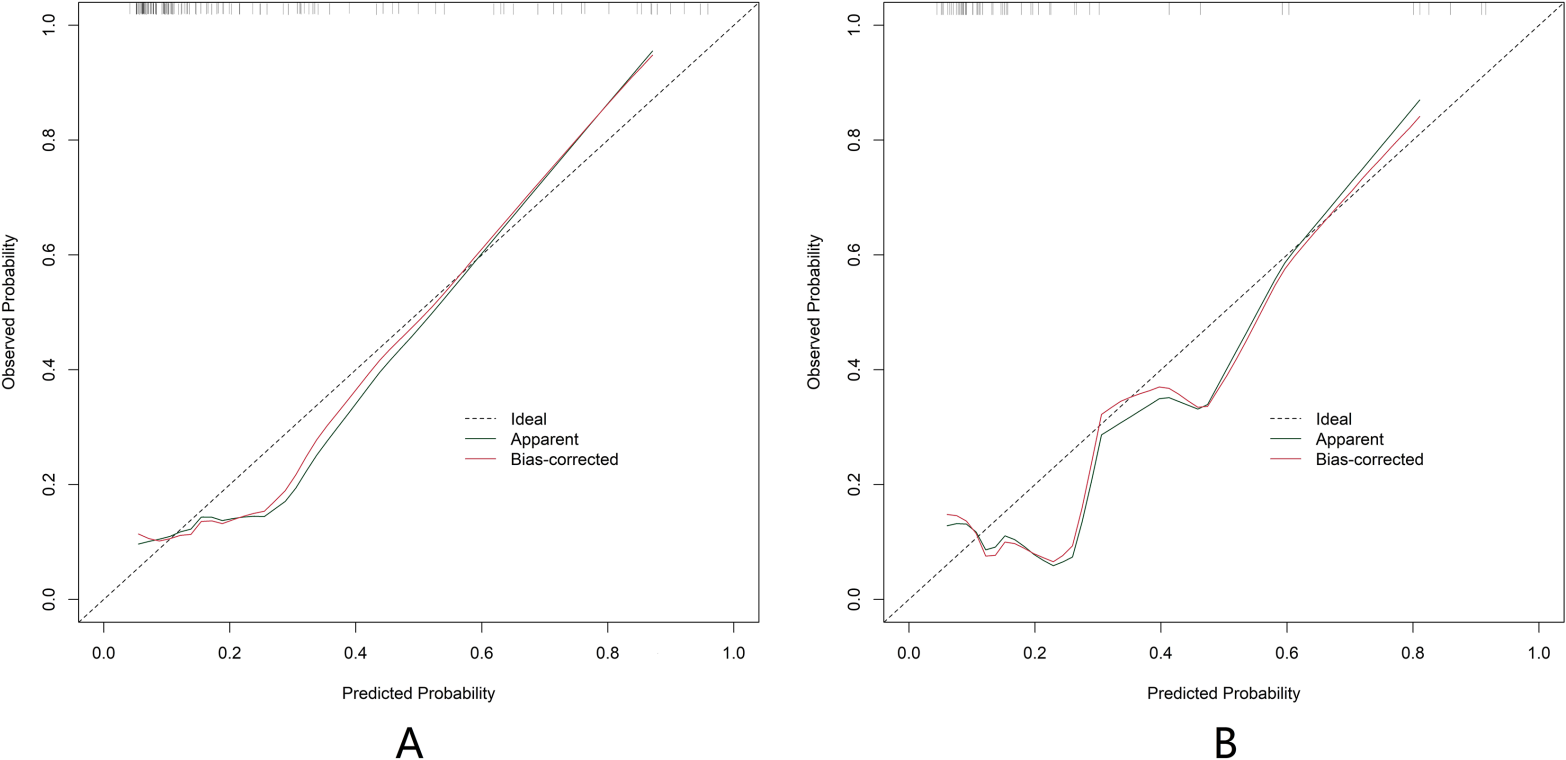
Calibration curves of predictive model based on nomogram of bladder cancer postoperative recurrence [**(A)** the training set: n=196; **(B)** the validation set: n=84].

**Figure 4:**
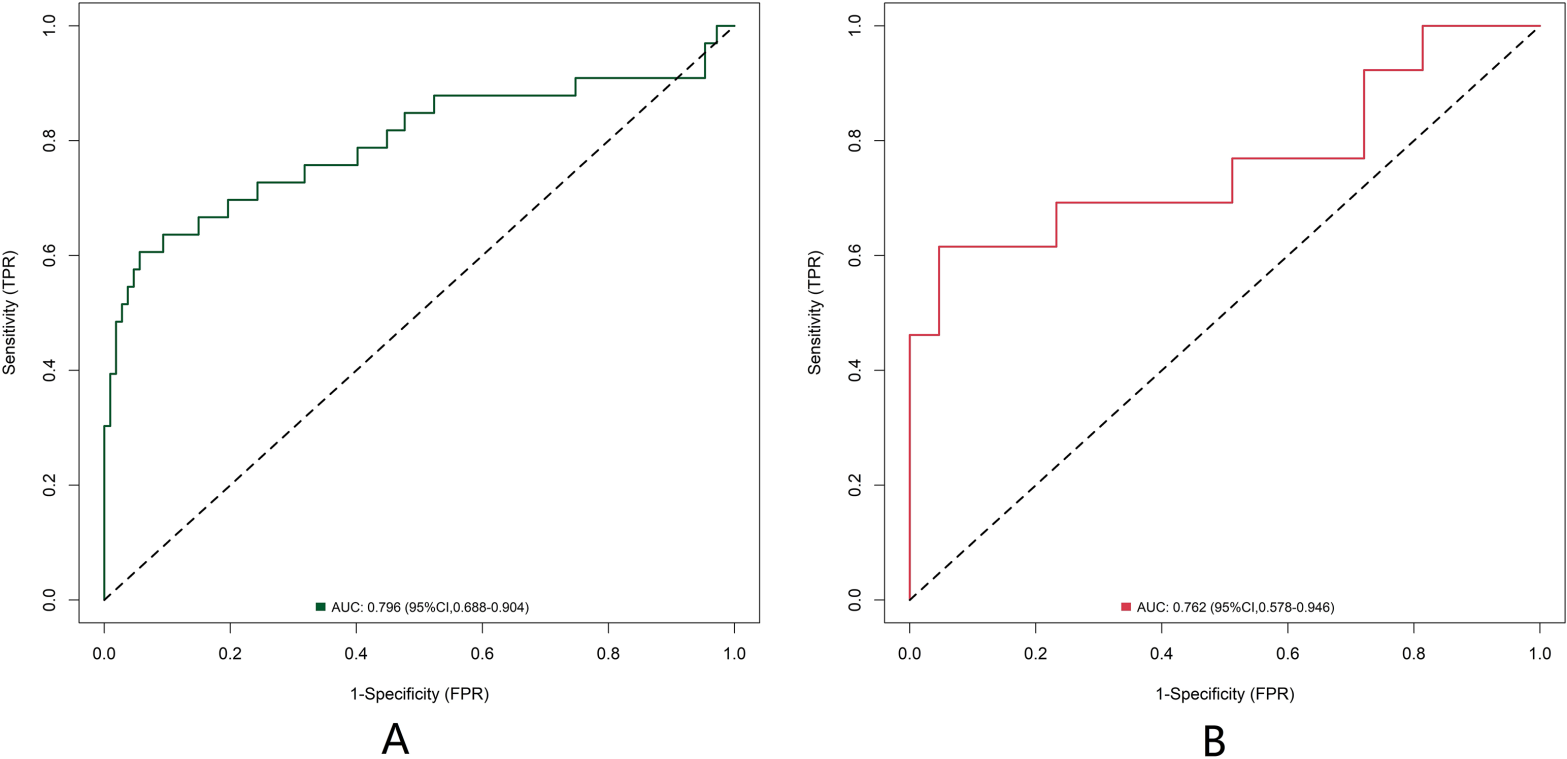
Receiver operating characteristic curves of predictive model based on nomogram of bladder cancer postoperative recurrence [**(A)** the training set: n=196; **(B)** the validation set: n=84].

In the training and validation sets, the nomogram model C-index was 0.796 and 0.762, respectively, the calibration curve showed the mean absolute errors of predicted and actual values were 0.120 and 0.121, respectively, and the Hosmer Lemeshow test results were *χ*^2^ = 10.64, *P* = 0.223 and *χ*^2^ = 12.72, *P* = 0.122 respectively (**Figure 3**). The ROC curves were displayed in the training and validation sets, and the AUC of the nomogram model for predicting BC postoperative recurrence was 0.796 (95% CI: 0.688-0.904) and 0.762 (95% CI: 0.578-0.946), respectively, with sensitivities and specificities of 0.606, 0.944 and 0.615 and 0.953 respectively (**Figure 4**).

### 3.6 Decision curve analysis of nomogram prediction model for postoperative recurrence of BC

**Figure 5:**
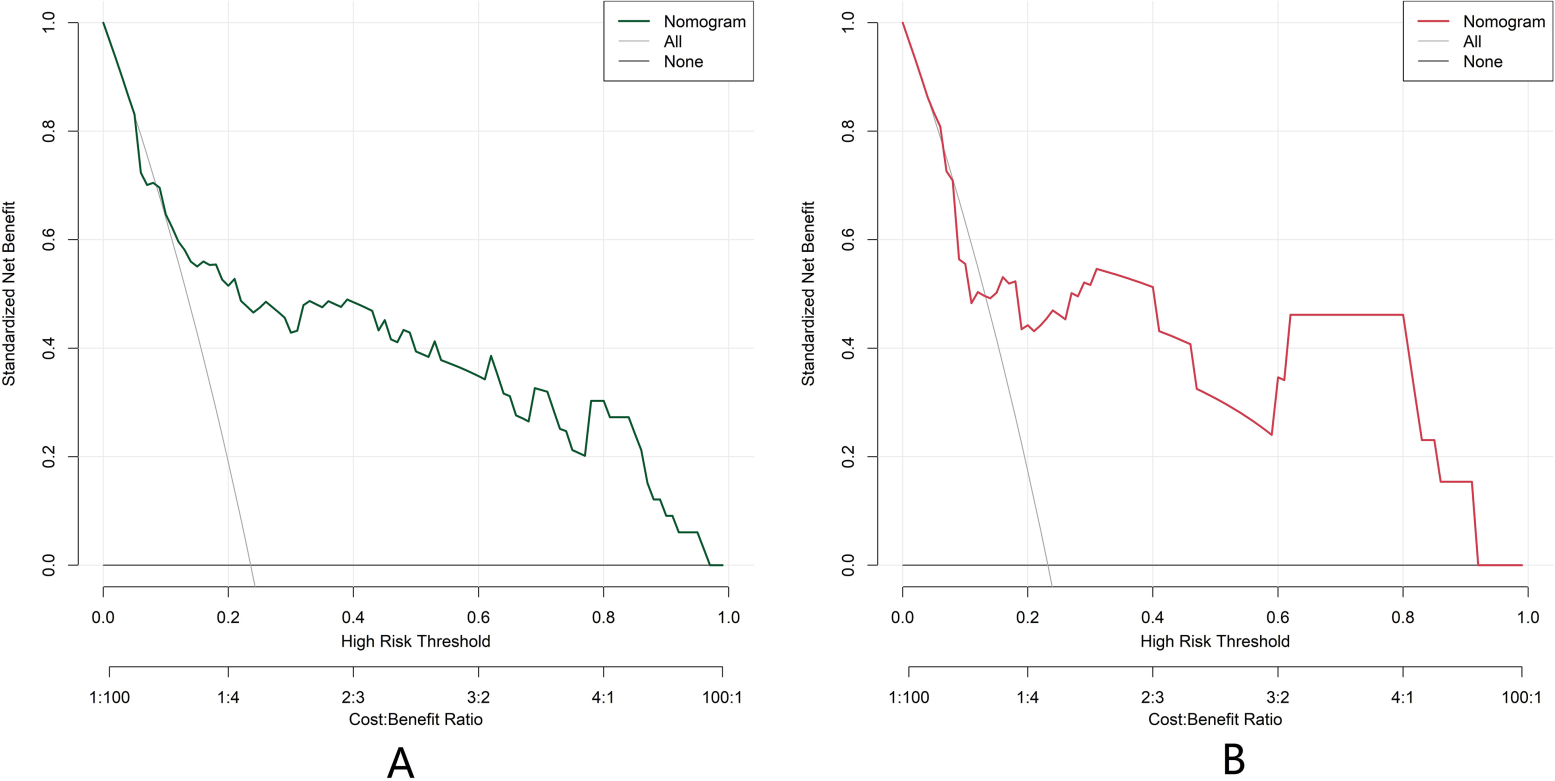
Decision curves of predictive model based on nomogram of bladder cancer postoperative recurrence [**(A)** the training set: n=196; **(B)** the validation set: n=84].

The decision curve shows that when the threshold probability is between about 0.05 and 0.95, the nomogram model constructed in this study has more clinical benefits in predicting the recurrence decision after BC surgery (**Figure 5**).

## 4 Discussion

Laparoscopic radical cystectomy procedure is an important surgical approach in the field of BC treatment. The principle is based on the minimally invasive advantage of laparoscopy. Laparoscopy instruments are placed through a small incision. With the help of the high-definition imaging system of laparoscopy, doctors can accurately and visually operate the bladder and the surrounding tissues to achieve complete resection of tumor lesions. In the bladder resection, the damage to the surrounding normal tissues can be reduced to the maximum extent, and the risk of bleeding during the operation is reduced. At the same time, some body functions are preserved (15). In terms of application status, the technique is currently widely used in major medical institutions, especially for early BC patients. Compared with traditional open surgery, it shortens the hospital stay of patients after surgery, reduces postoperative pain, facilitates the rapid recovery of patients, and improves the quality of life of patients. However, for some locally advanced or complex cases, the difficulty and risk of the operation are still high, and the technical requirements are more demanding (16, 17).

In order to further explore the factors affecting the recurrence of laparoscopic radical cystectomy procedure, multiple key indicators were included in this study through single-factor and multi-factor analysis. The first is the miR-18a and miR-122 in serum micro-RNA (miRNA). At the molecular mechanism level, serum miR-18a can regulate multiple signaling pathways closely related to tumor progression, such as PI3K-Akt and MAPK, and the abnormal activation or inhibition of these pathways is closely related to the invasion and metastasis potential of tumor cells (7, 18). MiR-122 can target and bind BC-related genes, and regulate the biological behavior of BC cells such as proliferation rate and migration ability by affecting the gene transcription and translation process (10).

As a common tumor marker, CEA serum concentration will be changed in the process of the development of a variety of tumors (19). After BC surgery, the increased serum CEA level may reflect the increased tumor cell load *in vivo*, suggesting that the tumor cells have residual or recurrent signs (20). The principle is that tumor cells secrete CEA into the blood circulation. When the tumor is in the stage of active growth, metastasis and recurrence, the secretion amount will be significantly increased (21). CA19–9 is widely used in the monitoring of digestive system tumors (22). Its significance in urothelial carcinoma is also being explored. After BC surgery, the interaction between tumor cells and the surrounding stromal cells may lead to an increase in CA19–9 release, as evidenced in studies of upper tract urothelial carcinoma (23), suggesting active tumor proliferation and changes in the tumor microenvironment.

Postoperative antibiotic use duration was also considered as a routine factor. Rational use of antibiotics after surgery is designed to prevent infection, but prolonged or short use may have adverse effects. Prolonged use may, on the one hand, trigger the breeding of drug-resistant bacteria, increase the risk of subsequent infection of patients, lead to immune dysfunction of the body, and indirectly create conditions for tumor recurrence; On the other hand, some antibiotics may interfere with the normal cell metabolism of the body and affect the anti-tumor immune mechanism (24).

Based on the above key factors, a Nomogram model for predicting postoperative recurrence of BC was constructed. This model integrated the independent risk factors of preoperative serum miR-18a and miR-122 levels, postoperative serum CEA and CA19–9 levels, and postoperative antibiotic use time. Through internal validation, both the training set and the validation set, the model shows good prediction performance. Various evaluation indicators, such as model discrimination reflected in the C-index index, the goodness of fit between the predicted value and the true value presented in the calibration curve, and the sensitivity and specificity shown in the ROC curve, all strongly proved the accuracy and reliability of the model, highlighting its potential application value in clinical practice. To illustrate the application of the nomogram, consider a hypothetical patient with the following characteristics: a preoperative serum miR-18a level of 10 (assigned approximately 35 points), a preoperative miR-122 level of 5.0 (25 points), a postoperative CEA level of 4.5 ng/mL (50 points), a postoperative CA19–9 level of 25 U/mL (40 points), and an antibiotic use duration of 7 days (60 points). The total points for this patient would be calculated as 35 + 25 + 50 + 40 + 60 = 210 points. Locating 210 points on the ‘Total Points’ axis and projecting down to the ‘Risk of Recurrence’ axis indicates a predicted recurrence probability of approximately 75% for this patient.

However, this study also has some limitations. Firstly, we exclusively included patients who did not receive neoadjuvant chemotherapy (NAC). While this selection was made to establish a model based on the natural postoperative course without the confounding effects of preoperative systemic therapy, it limits the generalizability of our findings to the broader population of muscle invasive bladder cancer patients for whom NAC is a standard, recurrence-reducing care. Therefore, at this stage, this model may be most applicable to patients with muscle-invasive bladder cancer who do not receive neoadjuvant chemotherapy because of contraindications or to patients with high-risk non-muscle-invasive bladder cancer, and its generalizability needs to be further validated in a broader population. Secondly, it exclusively included patients who underwent ileal conduit or orthotopic neobladder reconstruction, thereby excluding those who received ureterocutaneostomy. Ureterocutaneostomy is a valid clinical option, typically reserved for patients with high surgical risk and poor overall condition. The decision to focus on intestinal diversions was made to establish the prediction model based on a more homogeneous cohort in terms of surgical complexity and postoperative recovery. However, this choice limits the generalizability of our nomogram, and its applicability to the specific subgroup of patients undergoing ureterocutaneostomy, who may have a different risk profile, remains unknown and requires future validation. Thirdly, the measurement of CA19–9 and CEA was based on a single post-operative time point. While this reflects a common clinical scenario, it precludes analysis of their dynamic kinetics, which may provide superior predictive information for recurrence compared to a static value. Importantly, we acknowledge that the levels of these biomarkers can be influenced by adjuvant chemotherapy. Future prospective studies designed with protocol-defined serial blood draws are needed to establish the optimal post-operative trajectory of these biomarkers and to validate their role in a setting where chemotherapy effects are rigorously accounted for. Regarding the sample size, although the inclusion of 280 patients provided the initial data basis for model construction, in the complex and volatile field of tumor research, especially given the high heterogeneity of BC, the relatively limited sample size could not cover all possible scenarios, and expanding the sample size is expected to further improve the stability and accuracy of the model. At present, this study focuses on serological indicators and a few routine clinical indicators, which fail to fully incorporate such deep-seated factors as the tumor microenvironment and the patient’s genetic background. In addition, no external validation was performed due to limitations of current research resources and conditions. We did not perform a subgroup analysis to compare recurrence rates between pure urothelial carcinomas and histological variants (e.g., squamous cell carcinoma, adenocarcinoma). This was primarily due to the limited number of cases with variant histology in our cohort, which would have precluded a statistically meaningful comparison. Consequently, the predictive performance of our nomogram across different histological subtypes remains to be further validated in larger, specifically designed studies. Finally, our model relies on preoperative variables, including biopsy-based pathology, which may not reflect the final postoperative staging (e.g., disease upstaging). This inherent limitation of preoperative models should be considered when interpreting our predictions. Furthermore, the study design focused on baseline samples, preventing assessment of postoperative miRNA dynamics. Future prospective studies with longitudinal sampling are needed to evaluate the potential of miRNAs in monitoring recurrence.

In summary, the nomogram model based on single postoperative measurements of serum miR-18a and miR-122 suggests potential prognostic value for predicting recurrence in BC patients within the context of this study. Its clinical utility, however, must be interpreted with caution given the static nature of the measurement and the potential confounding influence of adjuvant chemotherapy. Future prospective studies incorporating serial biomarker measurements and detailed treatment data are needed to validate and refine this model for dynamic risk assessment.

## Data Availability

The raw data supporting the conclusions of this article will be made available by the authors, without undue reservation.
